# Elevated arterial blood pressure and body mass index among Nigerian preschool children population

**DOI:** 10.1186/1471-2431-14-64

**Published:** 2014-03-04

**Authors:** Odutola I Odetunde, Emeka E Neboh, Josephat M Chinawa, Henrietta U Okafor, Oluwatoyin A Odetunde, Osita U Ezenwosu, Uchenna Ekwochi

**Affiliations:** 1 Pediatric Nephrology Unit, Department of Pediatrics, University of Nigeria Teaching Hospital, UNTH, PMB 01129 Ituku-Ozalla Enugu State, Nigeria; 2 Cardiology Unit, University of Nigeria Teaching Hospital, UNTH, PMB 01129, Ituku-Ozalla, Enugu State, Nigeria; 3 Oncology and Hematology Unit, University of Nigeria Teaching Hospital, UNTH, PMB 01129, Ituku-Ozalla, Enugu State, Nigeria; 4 Department of Medical Biochemistry, College of Medicine, Enugu State University of Science and Technology (ESUT), Enugu, Nigeria; 5 Pediatric Surgery Unit, Department of Surgery, Enugu State University Teaching Hospital, Enugu, Nigeria; 6 Department of Pediatrics, Enugu State University Teaching Hospital, Enugu, Nigeria

**Keywords:** Hypertension, Pre-hypertension, Body mass index (BMI), Preschool children, Nigeria

## Abstract

**Background:**

Arterial blood pressure tends to rise with growth and development. Elevated blood pressure (EBP) in children usually occurs during the first two decades of life, and the children with hypertension tend to grow into adulthood with the high level of blood pressure. The prevalence of hypertension in children is increasing, the causes likely to be of different combination of factors. In this study we ascertained the prevalence of EBP in pre-school children in Enugu metropolis, South-East Nigeria and also determined its association with some factors like the Body Mass Index (BMI), urinalysis finding, family history, gender, age and socioeconomic class.

**Method:**

A Stratified method of sampling was used to select subjects from registered nursery schools (Pre- elementary school) within Enugu metropolis. Physical examination of the recruited pupils was done with emphasis on arterial blood pressure, anthropometric measurements and urinalysis.

**Result:**

Six hundred and thirty children (630) were studied out of which 345 (54.8%) were males and 285 (45.2%) were females. Sustained EBP (mainly systolic) were recorded in 12 pupils (1.9%) giving a prevalence of 1.9% of the pre-school population. The twelve (1.9%) pupils were all 5 years of age (p value = 0.001) and 11 (1.72%) of them were of under-weight BMI. The prevalence of obesity is 0.5% and that of under-weight is 92% of the studied population. There is no association between EBP and obesity (p value = 0.679). All the pupils with EBP had protein-free urine and no hematuria.

**Conclusions:**

EBP and under-weight malnutrition is common in children in 5 years age group. EBP in preschool children is not influenced by their body mass index, urinalysis finding, gender, family history of hypertension or socioeconomic class.

## Background

The prevalence of hypertension in children is on the increase [1], as blood pressure increases with growth and development and results in hypertension during the first two decades of life [2]. Children with elevated blood pressure tend to maintain that level of blood pressure into adulthood [3]. Early detection with effective control is essential so as to minimize cardiovascular complications in adulthood. The risk factors for hypertension includes obesity, family history of hypertension, change in dietary habits, decrease in physical activities and increasing stress as affected by the socioeconomic status [4]. The prevalence of hypertension in children varies between studies and depends on the studied age groups and the definition of hypertension in the studied population. Hypertension is defined as systolic or diastolic blood pressure greater than or equal to the 95th percentile for age and gender measured on at least three separate occasions and pre- hypertension as systolic or diastolic blood pressure between 90th and 95th percentile [5].

In children, systemic hypertension is uncommon with only about 1 percent of the childhood population having blood pressure significantly above the normal range [6]. Several studies on prevalence and risk factors for hypertension like high Body mass index (BMI) have focused more on older children and adolescents with less attention on the pre-school age children [7-11]. It is assumed that abnormal BMI in pre-school age children may not be related to medical problem and that health problem may not emerge until a child is overweight for several years (probably till adolescent age) before the evolution of obesity and its related health problem [12].

In this study we evaluated the usefulness of simply screening for high blood pressure and BMI calculation in early detection of blood pressure disorders in healthy pre-school children in Enugu metropolis in southeast Nigeria, with the objectives of determining the prevalence of pre hypertension and hypertension in these children and to ascertain its relationship with some known risk factors in asymptomatic subjects.

## Methods

### Setting

This study was carried out in Enugu metropolis, the capital city of Enugu State south-east of Nigeria. The population of the preschool age group in Enugu town was 37,556 (7 percent of total population) [13]. Written informed consent was obtained from the parents or the caregivers of the subjects before being enrolled into the study. The Ethical committee of the University of Nigeria Teaching Hospital (UNTH) gave approval for the study and permission was also obtained from State Ministry of Education before the commencement of the study.

### Recruitment of subjects

A Stratified method of sampling was employed to get a sample that represents the population. The three local government areas (Enugu north, Enugu east and Enugu south) were used as the strata. In each stratum (local Government Area) four nursery schools were selected randomly from a constructed sampling frame of 75 Nursery schools.

In each of the nursery schools, a sample size of 50–60 pupils was calculated and were selected randomly based on population of the school and were given the proforma with universal sample bottle for urine collection. Thus, the numbers of proforma given per-school depend on the population of the school. Selection of schools was influenced by the cooperation and readiness of the management to participate in the research. Six hundred and thirty (n = 630) subjects gave their informed consent to be part of the study and also met the inclusion criteria of relatively healthy children for the study. Exclusion criteria include subjects with age less than 2 and greater than 5 years old (at the last birthday), presence of symptoms of renal disease like dysuria, increased urinary frequency or urgency, macroscopic hematuria and facial or pedal edema, Presence of fever a week prior to the study, Lack of parental or guardian consent.

We carefully made sure that confounding and modifying factors of protenuria and hematuria such as fever, intense activity or exercise, dehydration, emotional stress were ruled out in our study. This was done with thorough history taking and physical examination.

## Methods

Physical examinations which included blood pressure, height, weight, pulse rate, and temperature measurements were carried-out on the subjects by trained physicians in the research team coordinated by the principal author in a standardized way. Emphasis of the physical examinations was on Body Mass Index (BMI) and Blood Pressure (BP) measurement. The BMI was calculated by the formular: BMI = Weight (Kg)/height (M^2^). We used the World Health Organization and Centers for Disease Control to classify BMI into underweight as BMI less than the 5th percentile, healthy weight as BMI of 5th up to the 85th percentile, overweight as BMI of 85th to less than the 95th percentile and obese as BMI equal to or greater than the 95th percentile for age and gender [14,15]. The blood pressure was measured by one observer. Seven or nine centimeter wrap-around cuffs were used depending on which width most closely approximated two thirds of the length of the upper arm. A mercury sphygmomanometer and an open-bell side of pediatrics-size Rappoport stethoscope were employed. Readings were recorded from right arm of the pupil in sitting position at the end of the routine examination and if the pupil was crying, the blood pressure was not taken until he or she became calm. The determined systolic and diastolic pressures were ascertained from three readings using the mean value of the three. An interval of 60 minutes was allowed and the cuff was completely deflated between readings. The systolic reading was taken as the first korotkoff-sound and the diastolic pressure was taken as the point where complete disappearance of the sound occurred (onset of 5th korotkoff-sound). We used the National High Blood Pressure In Children and Adolescent (2004 Working Group) guideline for diagnosis and Classification to classify the subjects into Normal BP with reading less than 90th percentile, Pre-hypertension with BP more than 90th percentile but less than 95th percentile while Hypertension was considered in subject with average reading greater than or equal to 95th percentile for age, gender, weight and height measured in at least three separate occasion [5].

We ensured stability of all our measurements through a constant reading of all our measurements that when other researchers measured same parameters, the error margin was insignificant. This is called “inter-rater reliability” or “inter-rater agreement [16]”.

Early morning urine specimens were collected from the subjects with sustained elevated BP and the completed proforma. The urine specimen was divided into 2 aliquots of 2 mls and 10 mls. The urinalysis was done on the uncentrifuged first aliquot (2 mls) of urine specimen using Combi 9 test strips by one of the authors. The second aliquot of the urine specimen and urine specimen of subjects with abnormal result (proteinuria and/or haematuria) were then transferred to the 10 ml test tubes, and were serially labeled and sent for microscopy. The specimens were centrifuged at 2000 rpm for 5 minutes. The microscopy was done on the sediments to determine presence of cast, red blood cells, white blood cells and crystals. From the proforma filled by the parents or care givers the family history of hypertension and renal disease was retrieved.

The families were assigned socioeconomic classes using the recommended method (modified) by Oyedeji [17]. The parents’ occupation and highest education attained were scored from 1 (highest) to 5 (lowest). The mean score for both parents gives social class falling within the 1–5 range. Those with the mean score of < 2 were further reclassified into upper class while those with the mean score of >2 were reclassified into lower social class. According to the protocol, for the occupation score, those in upper social class included parents, such as senior public officers, large-scale traders, large-scale farmers and professionals. Lower class included artisans, primary school teachers, peasant farmers, labourers and the unemployed. For the education score, those with PhD, master degree, bachelor degree and higher national diploma (HND) were categorized as upper class. Those with ordinary national diploma (OND), national certificate of education (NCE), technical education, grade II teachers’ certificate, junior and senior secondary school certificate, primary school certificate and those with no formal education were classified as lower social class [17].

### Statistical analysis

Data analysis was done using statistical package for social sciences (S.P.S.S for windows 17.0 output). Frequencies were compared using Chi squared test. Chi square was also used to test for association between differences in proportions of the independent variables (BMI, Age, Gender, Urine analysis findings and Family background) on the dependent variables (Blood Pressure reading). All the continuous variables were re-grouped into categorical forms (e.g. BMI was regrouped as Under Weight, Healthy Weight, Over-Weight and Obese) also, age has its categories as age two, age three, age four and five. Sex was grouped as Male and female, other categorical variables include family history of blood pressure and family history of renal diseases grouped as ***YES*** for those with history of renal diseases and high blood pressure in their family, and ***NO*** for those who have no history of renal diseases and high blood pressure in their family. P value of < 0.05 was considered significant. Data presentation was in tables.

## Results

A total of 630 subjects were studied comprising of 345 males (54.8%) and 285 females (45.2%), giving a male: female ratio of 1: 0.8. Of the 345 male subjects, 66 (19.1 percent) were aged 2 years; 85 (24.7%) 3 years; 95 (27%) 4 years and 99 (28.7%) 5 years. On the other hand, of 285 female subjects, 55 (17.5%) were aged 2 years; 84 (29.5%) aged 3 years; 68 (23.9%) aged 4 years and 83 (29.1%) aged 5 years. Sustained elevated BP were recorded in 12 (1.9%) of the studied population nine (1.4%) of which had pre -hypertension and 3 (0.5%) hypertension (Table 1). The twelve (1.9%) subjects with elevated BP were aged 5 years (P value = 0.001) (Table 1). Of the twelve subjects with elevated blood pressure 8 (1.3%) were male while other 4 (0.6%) were females (Table 1). Three (0.5%) of the subjects were Obese, 2 (0.3%) were over-weight while 581(92.2%) were underweight. Elevated BP has no association with obesity or over-weight (Table 1). The variation of result within the social classes was not significant (P value = 0.56) (Table 2).

**Table 1:** Showing percentage distribution of children by the level of blood pressure in relation to their age, gender and BMI

**Percentage distribution of the children by the level of their blood pressure**				
Blood pressure (BP)	Frequency (N)		Percentage (%)	
Hypertension	3		0.5	
Pre- Hypertension	9		1.4	
Normal BP	618		98.1	
Total	630		100	
**Distribution of elevated blood pressure by age of the children**				
Age (Yrs)	Pre HBP	HBP	Normal	Total
2 - < 3	0 (0)	0 (0)	116 (18.4)	116 (18.4)
3- < 4	0 (0)	0 (0)	169 (26.8)	169 (26.8)
4 - < 5	0 (0)	0 (0)	163 (25.9)	163 (25.9)
5	9 (1.4)	3 (0.5)	170 (27.0)	182 (28.9%)
Total	9 (1.4)	3 (0.5)	618 (98.1)	630 (100)
***χ***^**2**^ **= 28.76 df = 5 P- value = 0.001**				
**Distribution of elevated blood pressure by gender of the children**				
Gender	Pre HBP	HBP	Normal	Total
Male	6 (0.95)	2 (0.32)	337 (53.5%)	345 (54.8)
Female	3 (0.45)	1 (0.18)	281 (44.6)	285 (45.2)
Total	9 (1.4)	3 (0.5)	618 (98.1)	630 (100)
***χ***^**2**^ **= 0.70**	**df = 2 P-value = 0.71**			
**Distribution of elevated blood pressure by BMI of the children**				
BMI Classification	Pre HBP	HBP	Normal	Total
Underweight	9 (1.4)	2 (0.32)	570 (90.5)	581 (92.2)
Healthy weight	0 (0)	1 (0.18)	43 (6.8)	44 (7)
Overweight	0 (0)	0 (0)	2 (0.3)	2 (0.3)
Obese	0 (0)	0 (0)	3 (0.5)	3 (0.5)
Total	9 (1.4)	3 (0.5)	618 (98.1)	630 (100)
***χ***^**2**^ **= 3.98 df = 5**	**P- value = 0.68**			

**Table 2:** Distribution of subjects with elevated blood pressure by socio-economic class

	**Blood presure**		
**Class**	**High**	**Normal**	**Total**
	**n (%)**	**n (%)**	**n (%)**
Upper	2 (2.1%)	93 (97.9)	95 (100)
Middle	7 (2.0%)	436 (98.0)	443 (100)
Lower	3 (3.2%)	89 (96.8)	92 (100)
Total	12 (1.9)	618 (98.1)	630 (100)
*χ*^2^ = 1.18	df = 2 P-value = 0.56.		

There is positive family of hypertension in 71 (11.3%) of the subjects out of which only 2 (0.3%) had elevated BP (Table 3). None of the subjects with elevated BP had positive family history of renal disease.

**Table 3:** Family history of high blood pressure and renal disease of the children by blood pressure

	**Blood pressure**			
Family history of Hypertension	Pre HBP	HBP	NORMAL	TOTAL
	n (%)	n(%)	n (%)	n (%)
Yes	1 (0.18)	1 (0.18)	69 (11)	71 (11.3)
No	8 (1.22)	2 (0.32)	549 (87.1)	559 (88.7)
TOTAL	9 (1.4)	3 (0.5)	618 (98.1)	630 (100)
*χ*^**2**^ = 1.5		df = 2	P-value = 0.5	
**Family history of Renal disease**	Pre HBP	HBP	NORMAL	TOTAL
	n (%)	n (%)	n (%)	n (%)
Yes				
No	8 (1.22)	2 (0.32)	549 (87.1)	559 (88.7)
TOTAL	9 (1.4)	3 (0.5)	618 (98.1)	630 (100)
*χ*^**2**^ = 1.5		df = 2	P-value = 0.5	

All the subjects with elevated BP had protein-free urine and no hematuria. Hypertension was observed in all the social classes of the studied population.

## Discussion

There are several technical challenges in comparing various prevalence studies on elevated arterial BP in children because of the dissimilarity in the studied population and criteria used in defining the condition. In this study, we employed the definition of elevated BP based on the 2004 National High Blood Pressure Education Program Working Group in Children and Adolescent to evaluate and classify our subjects [6]. Our studied population was healthy preschool age children in which the prevalence of elevated BP (systolic) of 1.9% was obtained, 1.4% and 0.5% for pre-hypertension and hypertension respectively. There are paucity of studies on elevated BP and risk factors in the preschool age children [18,19]. The prevalence of elevated BP in this study is lower when compare with 5.2% and 13% obtained by Vitolo et al. and William et al. respectively in their studies of a similar age population though in different settings [18,19]. The prevalence of 1.9 percent in this study is slightly higher when compared with that of 1% by National High Blood Pressure Education Program Working Group in Children in the United State of America [6]. In this study, subjects with hypertension were limited to children in 5 years age group with no gender preponderance. This is similar to the observation of Still and Cottom [20] who noted higher incidence of hypertension in the age group 5 to 10 years with no gender preponderance. The study was carried out in a metropolitan city where due to the challenges of low acceptability of exclusive breastfeeding initiative, high acceptability of breast milk substitutes and modernization of diet in the general population, majority (92.2%) of the subjects were underweight while only 7% had healthy weight.

The prevalence of obesity and overweight were 0.5% and 0.3% respectively and there were no association between these and elevated BP. Several studies in the general population in Nigeria have documented prevalence rate of obesity in children and adolescents to range from 0.3 – 18% [21-23] and Rosnner et al. in the study of 47,000 children reported that the risk of elevated blood pressure is higher in children in upper deciles of BMI compared with odd ratio of systemic hypertension ranging from 2.5 -5.7 mmHg [24]. Our finding of prevalence for obesity and overweight are in contrast with the report by Williams et al. [18] in a similar population though in an industrialized country where they documented a prevalence of 15% and 17% for obesity and overweight respectively while 13% had elevated BP. Virtually all the subjects with EBP in our study were of underweight BMI. This is in consonant with the findings of Sawaya AL et al. [25] in their study done in another developing country. They reported that the prevalence of hypertension in undernourished preschool children and those that recovered from under nutrition to be higher than that of the controls with normal nutrition and concluded that this may possibly impact on the morbidity and mortality related to hypertension during adulthood. Nearly all our subjects (92.2%) had underweight BMI. This may be a reflection of nutritional deficiency during prenatal life that continues in the post-natal period which is very common in the developing countries [25].

In this study, no association was observed between hypertension, proteinuria or hematuria which are surrogate markers for renal disease. The distribution of proteinuria and hypertension in this study had no significant preponderance for any particular socioeconomic group, although some of the renal disorders in Africa are related to infection and infestation which are commoner in the lower social class. There were also no signification association between EBP and positive family history of hypertension or renal disease in the subjects. Several studies had reported positive family history of hypertension, renal disease, change in dietary habits, decrease physical activities and increasing stress as affected by the socioeconomic status as risk factors for development of EBP in children [4,18,19].

## Conclusions

There is a significant association between EBP and pupils in 5 years age group while obesity, urinalysis finding, gender, family history and socioeconomic class do not affect BP.

Pre-admission health examination should be introduced at primary school level and should include BP and BMI measurement in order to identify those with EBP. All cases of elevated blood pressure with or without abnormal BMI detected during this screening should be referred to Pediatric Nephrologists for proper evaluation and follow-up.

## Abbreviations

BMI: Body mass index; EBP: Elevated blood pressure.

## Competing interests

The authors declare that they have no competing interests.

## Authors’ contributions

OIO, EEN, JMC and OUE envision the study, participated in its design, data collection and coordination of the study and drafted the manuscript. HUO, JMC, OAO and UE contributed substantially to data collection, analysis and interpretation and revision of the manuscript. OIO, EEN, OAO, HUO, JMC, UE and OUE did the final revision of the manuscript. All authors read and approved the final manuscript for publication.

## Pre-publication history

The pre-publication history for this paper can be accessed here:

http://www.biomedcentral.com/1471-2431/14/64/prepub
